# The Effect of Exercise Training on Gait, Balance, and Physical Fitness Asymmetries in Persons With Chronic Neurological Conditions: A Systematic Review of Randomized Controlled Trials

**DOI:** 10.3389/fphys.2020.585765

**Published:** 2020-11-12

**Authors:** John W. Farrell, Jordan Merkas, Lara A. Pilutti

**Affiliations:** Interdisciplinary School of Health Sciences, University of Ottawa, Ottawa, ON, Canada

**Keywords:** multiple sclerosis, stroke, asymmetry, exercise, gait, balance

## Abstract

**Background:** Persons with chronic neurological conditions (CNCs) often present with asymmetrical impairments, creating significant differences between contralateral limbs in body functions. These asymmetries have been associated with reduced mobility and balance, and are often targeted for reduction during rehabilitation. Exercise training has established benefits for persons with CNCs, and may have positive effects on asymmetry outcomes.

**Objectives:** The purpose of this review was to summarize the current evidence for the effects exercise training on gait, balance, and physical fitness asymmetry in randomized control trials (RCTs) of persons with CNCs.

**Methods:** A search of four electronic databases (EMBASE, CINAHL, SPORTdiscus, and ovidMEDLINE) was conducted following the structured Preferred Reporting Items for Systematic Reviews and Meta-Analyses (PRISMA) guidelines.

**Results:** The search retrieved 3,493 articles, with 465 articles assessed for eligibly, and nine articles meeting the criteria for inclusion. Of the included articles, five incorporated resistance exercise, three incorporated aerobic exercise, and one incorporated combined exercise (i.e., resistance and aerobic exercise). Gait asymmetry improved significantly in four studies after resistance, aerobic, and combined exercise. Significant improvements in weight bearing asymmetry were reported in three studies after resistance exercise. One study reported significant improvements in both gait and balance asymmetry after resistance exercise.

**Conclusions:** Preliminary evidence suggests that exercise training, as a component of rehabilitation, may have positive effects on gait and balance asymmetry in persons with CNCs. Several limitations of the current literature were noted, including a limited number of studies, combination of exercise with other rehabilitation modalities, a lack of reporting on exercise prescriptions (e.g., number of repetitions, intensity), and variability in the calculation of asymmetry outcomes. These limitations prevent definitive conclusions on the effects of exercise training on asymmetry outcomes. Future trials are needed to determine the potential of exercise training for reducing asymmetry in persons with CNCs.

## Introduction

Chronic neurological conditions (CNCs) are among the leading causes of death and disability worldwide (Heymann et al., 2007). CNCs can be the result of an immediate event (i.e., ischemic stroke) or chronic progression (i.e., multiple sclerosis (MS), Parkinson's disease (PD), with both resulting in neurodegeneration, neurotoxicity, and inflammation within the central nervous system (CNS) (Zeiler and Krakauer, 2013). The accumulated damage impairs the propagation of action potentials to the peripheral tissues, resulting in the loss of central body structures and impairments in body functions (Ammann et al., 2014). Common impairments in this population include deficits in mobility, balance, and muscular strength. Impairments can be due to residual effects from an immediate event or can accumulate over time coinciding with disease progression (Clarke and Black, 2005; Hankey et al., 2007; Benedict et al., 2011; Cumming et al., 2013; DeLuca et al., 2015).

In persons with CNCs, impairments in body functions often present in an asymmetrical pattern, creating a significant difference between contralateral limbs and muscle groups (Rudroff and Proessl, 2018), often described as an “affected” and “non-affected” limb (Sun et al., 2018). Indeed, asymmetries have been observed for temporal and spatial gait outcomes, limb loading, and muscular strength and power in these populations (Djaldetti et al., 2006; Lauziere et al., 2014; Rudroff and Proessl, 2018). These asymmetries have been associated with reduced mobility and balance, gait inefficiencies, cumulative musculoskeletal injuries in the non-affected limb, loss of bone mineral density in the affected limb, and reductions in physical activity (Jørgensen et al., 2000; Patterson et al., 2008; Ellis et al., 2013). If not addressed through rehabilitation interventions, asymmetries may worsen over time due to the development of compensatory strategies that often exacerbate asymmetry, and may lead to greater functional impairments and reduced participation in daily life (Patterson et al., 2015).

Reducing impairment and restoring function (e.g., improving mobility and balance) is the predominate focus of rehabilitation programs for persons with CNCs. Due to reported associations between physiological fitness (e.g., cardiorespiratory fitness and lower limb strength) and mobility and balance outcomes, rehabilitation programs often incorporate exercise training into the overall program design (Sandroff et al., 2013). Indeed, improvements in cardiorespiratory fitness and muscular strength and power have been reported in this population in response to exercise training, in addition to improvements in gait, balance, and physical fitness (Motl and Sandroff, 2015; Hasan et al., 2016; Platta et al., 2016). Rehabilitation professionals often target reductions in asymmetry, particularly in gait outcomes, to improve overall mobility and balance (Teasell et al., 2003; Patterson et al., 2015). However, the efficacy of exercise training for reducing asymmetries in persons with CNCs, and the potential effects on fitness and functional outcomes remains unclear. As exercise becomes increasingly recommended as a key component of comprehensive disease management for persons with CNCs, it is critical that we understanding the full potential of this therapy.

The purpose of this review was to evaluate and summarize the current evidence for the effects of exercise training on asymmetry, specifically parameters of gait, balance, and physical fitness. We further characterized the effects of the interventions on overall physical fitness and function outcomes, and explored potential associations between changes in asymmetry outcomes and changes in physical fitness and function. The results of this review will provide a summary of the current evidence for exercise training as a therapeutic approach to reduce asymmetries in persons with CNCs, and will provide recommendations for future interventions.

## Materials and Methods

### Article Inclusion Criteria and Search Strategy

The current review aimed to summarize studies examining the efficacy of exercise training on asymmetry in physical fitness, physical function, and gait in persons diagnosed with either MS, PD, or having experienced an ischemic stroke. These conditions were selected given the presence of reported asymmetries in function within these groups (Djaldetti et al., 2006; Lauziere et al., 2014; Rudroff and Proessl, 2018). Exercise training was defined as any “planned structured and repetitive bodily movement done to improve or maintain one or more components of physical fitness” (Bouchard et al., 1994). The current review was conducted in accordance with the PRISMA (preferred reporting items for systematic review and meta-analyses) guidelines (Moher et al., 2009). A search of four electronic databases (EMBASE, CINAHL, SPORTdiscus, and ovidMEDLINE) using the search terms “central nervous system condition” OR “central nervous system disease” OR “stoke” OR “multiple sclerosis” OR “parkinson's disease” AND “asymmetry” or “bilateral” or “unilateral” AND “muscle” OR “strength” OR “walking” OR “gait” OR “posture” OR “postural balance” OR “balance” OR “movement” OR “physical mobility” OR “mobility” was conducted. The search strategy was developed in collaboration with a Health Sciences librarian and conducted in October of 2019 by JWF.

The inclusion criteria involved full text, English language articles that included: (1) participants with a diagnosis of stroke, MS, or PD; (2) a randomized controlled trial (RCT) study design; (3) an intervention involving an exercise training component; and (4) at least one asymmetry outcome related to physical fitness, physical function, or gait. Investigations that included exercise training in addition to other rehabilitation strategies as part of a comprehensive intervention were not excluded if they met all other inclusion criteria.

### Article Quality Assessment

The quality of each article was assessed using the Tool for the Assessment of Study Quality and Reporting in Exercise (TESTEX) (Smart et al., 2015). The TESTEX is a quality and reporting assessment tool designed specifically for use in exercise training studies, and includes assessing the reporting of all exercise training parameters (e.g., intensity, duration, frequency, and mode). This tool uses a 15-point scale, with 5 points for study quality and 10 points for reporting of exercise training parameters. Higher scores are indicative of greater study quality. Articles were evaluated independently by JWF and JM. Discrepancies resolved by re-examining the articles and through discussion.

### Descriptive Approach

Data were extracted relative to participants (e.g., disability status or level of ambulation), exercise training characteristics (e.g., modality), asymmetry outcomes, and the efficacy of the intervention on asymmetry outcomes, as well as physical fitness and physical function outcomes. Data were first extracted by JWF and then checked by JM. Data were categorized and summarized by the type of exercise training intervention, as either resistance, aerobic, or combined (resistance and aerobic) exercise. The number of studies that used each type of exercise training, as well as the number of studies reporting statistically significant changes in asymmetry, physical fitness, and physical function outcomes were summarized using descriptive statistics. Additionally, studies that reported associations between changes in asymmetry outcomes and changes in physical fitness and physical function outcomes were summarized using descriptive statistics. A meta-analytic approach was not attempted, given the limited number of studies retrieved.

## Results

**Figure 1:**
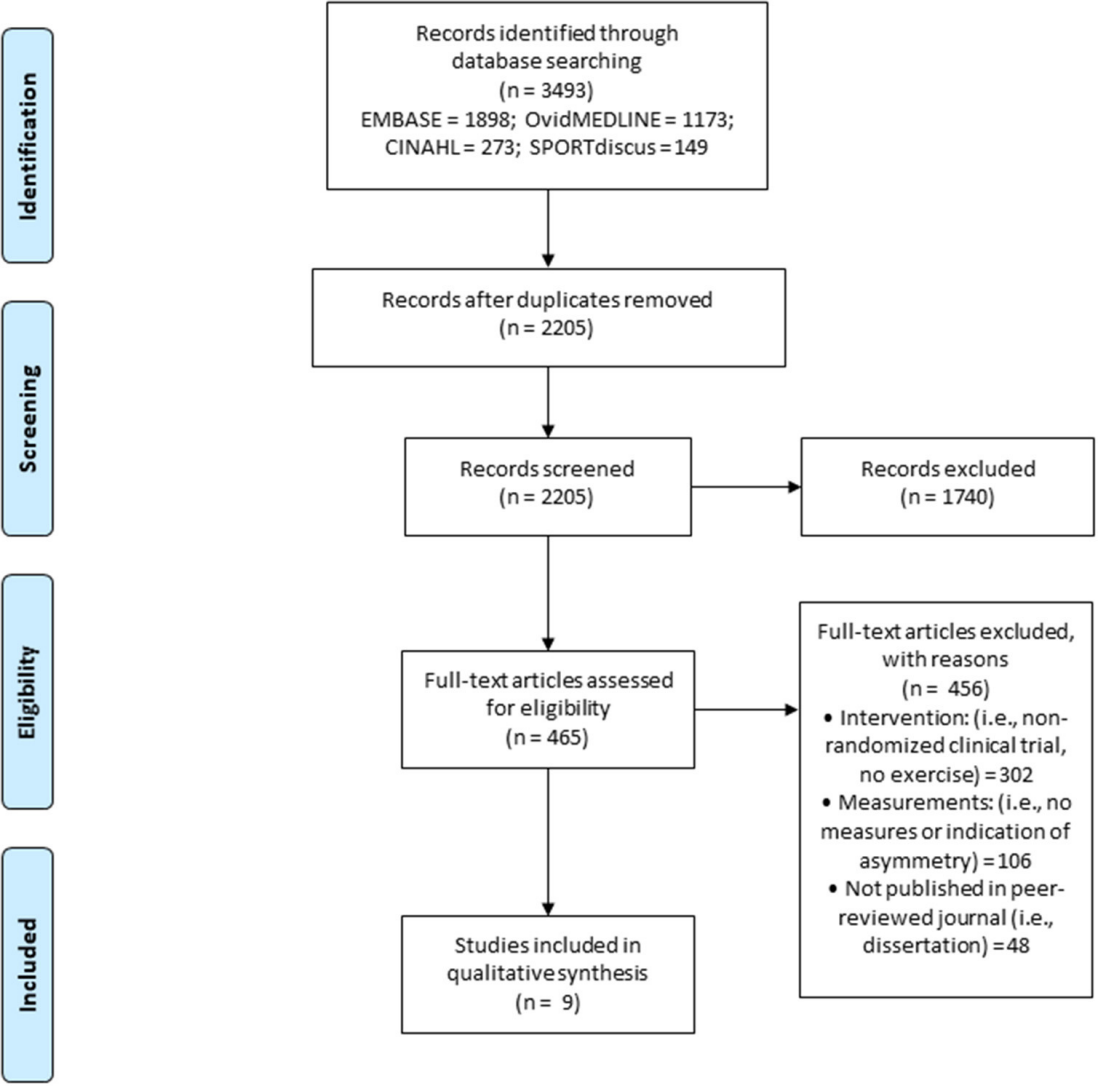
PRISMA (the preferred reporting items for systematic review and meta-analyses) flow diagram for the literature review process.

Figure 1 illustrates the literature search, article screening process, and specific reasons for article exclusion. The electronic database search retrieved 3,493 articles. After removal of duplicates, 2,205 articles remained. In total 2,196 did not meet specific inclusion criteria, leaving nine studies included in the review.

Studies were grouped based on the exercise training modality as resistance (*n* = 5), aerobic (*n* = 3), and combined (resistance and aerobic; *n* = 1) exercise. Table 1 summarizes the study, participant, and exercise training characteristics. Although the search included persons with stroke, MS, and PD, the articles retrieved yielded studies involving persons with stroke (*n* = 8) (Tung et al., 2010; Sungkarat et al., 2011; Kim et al., 2015, 2017; Liu et al., 2016; Sheikh et al., 2016; e Silva et al., 2017; Lewek et al., 2018) or MS (*n* = 1) only (Escudero-Uribe et al., 2017). Overall, the interventions were prescribed for 30–100 min/session, 2–6 sessions/week for 3–12 weeks. In addition to exercise training, other intervention components included constraint movement therapy (e Silva et al., 2017), task-oriented training (Kim et al., 2015), gait training (Sungkarat et al., 2011; Sheikh et al., 2016; Kim et al., 2017; Lewek et al., 2018), sit-to-stand training (Tung et al., 2010; Liu et al., 2016), balance training, and whole-body vibration training (Escudero-Uribe et al., 2017).

**Table 1:** Study, participant, and exercise training characteristics of the 9 articles reviewed, grouped by training modality as resistance, aerobic, and combined exercise.

**Study characteristics**		**Participant characteristics**				**Exercise training characteristics**		
**Reference (Quality)**	***n* (Ex 1; Ex 2; Con)**	**Age (y) Mean ± SD**	**Condition**	**Impairment level**	**Time since diagnosis (y) Mean ± SD**	**Duration (weeks)**	**Frequency (x/week)**	**Session length (min)**
**Resistance exercise (*****n*** **=** **5)**								
Liu et al., 2016	25; 0; 25	50.3 ± 11.5	Stroke	Brunnstrom motor recovery stage: 3	0.3 ± 0.1	4	5	30
Sheikh et al., 2016	14; 0; 14	56.9 ± 12.3	Stroke	Ashworth index: 2–5	3.0 ± 7.2	6	6	90
Kim et al., 2015	13; 0; 12	59.7 ± 9.4	Stroke	Ashworth index: <2	0.6 ± 0.4	3	5	30–50
Sungkarat et al., 2011	17; 0; 18	53.0 ± 9.3	Stroke	Orpington prognostic scale: 3.2–5.2	0.4 ± 0.4	3	5	60
Tung et al., 2010	16; 0; 16	51.9 ± 13.1	Stroke	NR	3.3 ± 1.2	4	3	30–45
**Aerobic exercise (*****n*** **=** **3)**								
Lewek et al., 2018	12; 14; 11	58.6 ± 12.2	Stroke	NR	3.3 ± 2.9	18 sessions	NR	30–35
e Silva et al., 2017	15; 0; 18	56.5 ± 10.0	Stroke	NR	3.0 (1–7) [median (IQR)]	9 sessions	NR	30
Escudero-Uribe et al., 2017	16; 14; 18	44 (range 22–62)	MS	EDSS: 1.5–4.5	8.6 ± 6.4	12	2	60–100
**Combined exercise (*****n*** **=** **1)**								
Kim et al., 2017	18; 19; 17	63.2 ± 9.8	Stroke	Brunnstrom motor recovery stage: 3-4	0.6 ± 0.3	5	2	45

*Ex 1, experimental group 1; Ex 2, experimental group 2; Con, control group; NR, not reported; MS, multiple sclerosis; EDSS, expanded disability status scale*.

Table 2 summarizes the efficacy of the interventions on asymmetry and symmetry outcomes grouped by exercise training modality, and describes the method of calculation for each outcome. Five studies reported gait asymmetry or symmetry outcomes (Kim et al., 2015, 2017; Escudero-Uribe et al., 2017; e Silva et al., 2017; Lewek et al., 2018), while two included balance asymmetry or symmetry outcomes (Tung et al., 2010; Liu et al., 2016) and two studies included both gait and balance asymmetry or symmetry outcomes (Sungkarat et al., 2011; Sheikh et al., 2016). No studies reported on asymmetry in physical fitness outcomes. Due to differences in outcome measures and calculation methods, it was not possible to report on mean changes for asymmetry and symmetry outcomes. However, absolute changes are reported in Table 2 for these outcomes.

**Table 2:** Summary of the effects of exercise training on asymmetry and symmetry outcomes, method of calculation, and the results of the 9 articles reviewed, grouped by training modality as resistance, aerobic, and combined exercise.

**References**	**Asymmetry and symmetry outcomes**	**Calculation**	**Δ Asymmetry and symmetry outcomes (EX 1, EX2, Con)**
**Resistance exercise (n** **=** **5)**			
Liu et al., 2016	Dynamic WB^*^^†^	Non-paretic side/paretic side	1.70, NA, 0.06
Sheikh et al., 2016	Static WB^†^ Swing time Stance time Step length Gait symmetry ratio	% of total body weight supported by paretic limb Larger swing time/smaller swing time Larger stance time/smaller stance time Larger step length/smaller step length [(Paretic swing time/paretic stance time)]/[(non-paretic swing time/non-paretic stance time)]	9.86, NA, 2.43 0.32, NA, 0.20 0.03, NA, −0.03 0.14, NA. 0.06 0.51, NA, 0.26
Kim et al., 2015	Gait symmetry ratio^*^^†^	[(Paretic swing time/paretic stance time)]/[(non-paretic swing time/non-paretic stance time)]	2.69, 2.87, 2.59
Sungkarat et al., 2011	Step length^†^ Single-support time^†^ Static WB^†^	|1- (affected side/unaffected side)| |1- (affected side/unaffected side)| % of total body weight carried by affected limb	1.03, NA, 0.22 0.14, NA, 0.01 9.20, NA, 0.70
Tung et al., 2010	Static WB^*^	% of total body weight supported by affected limb	4.9, NA, 1.7
**Aerobic exercise (*****n*** **=** **3)**			
Lewek et al., 2018	Step length^*^ Stance time	Paretic/non-paretic limb Paretic/non-paretic limb	−0.02, −0.06, −0.06 0.00, −0.01, −0.00
e Silva et al., 2017	Swing time	Paretic swing time/non-paretic swing time	0.02, NA, −0.21
Escudero-Uribe et al., 2017	Step length^*^^†^	Not provided	−1.50, −0.80, 0.40
**Combined exercise (*****n*** **=** **1)**			
Kim et al., 2017	Gait symmetry ratio^*^^†^	[(Paretic swing time/paretic stance time)]/[(non-paretic swing time/non-paretic stance time)]	−0.78, −1.9, −0.08

*Δ asymmetry and symmetry outcomes: absolute change in asymmetry and symmetry outcomes from pre to post; Ex 1, experimental group 1; Ex 2, experimental group 2; Con, control group; NA, not applicable; WB, weight bearing*;

* *, statistically significant within group difference; p < 0.05*;

† *, statistically significant between group difference, p < 0.05*.

The effects of the interventions on physical fitness and physical function are described by exercise training modality in Table 3. Physical fitness outcomes were included in two studies (Tung et al., 2010; Kim et al., 2015), both reported as lower limb strength. Physical function outcomes were reported in six studies (Tung et al., 2010; Sungkarat et al., 2011; Liu et al., 2016; e Silva et al., 2017; Kim et al., 2017; Lewek et al., 2018), with four studies assessing balance (e.g., Berg balance scale and static and dynamic weight bearing) (Tung et al., 2010; Sungkarat et al., 2011; Liu et al., 2016; e Silva et al., 2017), two assessing lower limb function (e.g., sit-to-stand and time-to-rise) (Tung et al., 2010; Liu et al., 2016), two assessing agility (e.g., Timed-Up-and-Go) (Sungkarat et al., 2011; e Silva et al., 2017), and two assessing mobility (e.g., 10-m walking test and comfortable gait speed) (Kim et al., 2017; Lewek et al., 2018). The effects of the interventions on asymmetry and physical fitness are described below by exercise training modality.

**Table 3:** Summary of the effects of exercise training on asymmetry, physical fitness, and physical function outcomes and results of the 9 articles reviewed, grouped by training modality as resistance, aerobic, and combined exercise.

		**Outcomes**		
**References**	**Physical fitness**	**Δ Physical fitness (EX 1, EX 2, Con)**	**Physical function**	**Δ Physical function (EX 1, EX 2, Con)**
**Resistance exercise (*****n*** **=** **5)**				
Liu et al., 2016	NA		Dynamic balance, CoP sway length (cm^2^)^*^^†^ Static balance, CoP sway area (cm)^*^^†^ Berg balance scale (points)^*^^†^ Time to rise (s)^*^^†^	84.24, NA, 67.74 27.85, NA, 21.95 8.40, NA, 5.80 0.90, NA, 0.42
Sheikh et al., 2016	NA		NA	
Kim et al., 2015	AS HF strength (lbs)^*^^†^ AS HE strength^*^^†^ AS KF strength^*^^†^ AS KE strength^*^^†^ AS DF strength^*^^†^ AS PF strength^*^^†^	18.40, 17.78, −4.93 5.04, 7.98, −0.21 2.21, 6.25, −0.17 5.21, 13.67, −0.12 6.95, 13.78, −0.41 5.02, 12.4, 0.22	NA	
Sungkarat et al., 2011	NA		Berg balance scale (points)^†^ Timed-up-go (s)^†^	9.47, NA, 3.41 9.88, NA, 4.41
Tung et al., 2010	AS HE strength (% of participant's body weight)^*^^†^ NAS HE strength^*^ AS KE strength^*^ NAS KE strength^*^ AS PF strength NAS PF strength^*^	3.1, NA, 3.2 2.6, NA, 0.1 4.1, NA, 5.1 4.3, NA, 4.7 2.5, NA, 3.0 4.1, NA, 2.4	Berg balance scale (points)^*^ Sit-to-stand (s)^*^^†^	3.50, NA, 2.80 −2.40, NA, 0.20
**Aerobic exercise (*****n*** **=** **3)**				
Lewek et al., 2018	NA		Comfortable gait speed (m/s)	0.12 (collapsed across all groups for analysis)
e Silva et al., 2017	NA		Berg balance scale^*^^†^ Timed-up-go (s)^*^^†^	3.6, NA, 5.3 −2.7, NA, −2.9
Escudero-Uribe et al., 2017	NA		NA	
**Combined exercise (*****n*** **=** **1)**				
Kim et al., 2017	NA		10-m walking test (m/s)	0.07, 0.17, 0.03

*Δ, change in outcome from pre to post; Ex 1, experimental group 1; Ex 2, experimental group 2; Con, control group; NA, not applicable; WB, weight bearing; HF, hip flexion; HE, hip extension; KF, knee flexion; KE, knee extension; DF, dorsiflexion; PF, plantarflexion; AS, affected side; NAS, non-affected side; lbs, pounds; m/s, meters per second; s, seconds; cm, centimeters*;

* *, statistically significant within group difference, p < 0.05*;

† *, statistically significant between group difference, p < 0.05*.

### Resistance Exercise Training

Investigations that included resistance exercise focused on improving lower limb strength. Three (Sungkarat et al., 2011; Kim et al., 2015; Sheikh et al., 2016) and four (Tung et al., 2010; Sungkarat et al., 2011; Liu et al., 2016; Sheikh et al., 2016) of the five studies reported asymmetry outcomes for gait and weight bearing, respectively. One of two studies (Kim et al., 2015; Sheikh et al., 2016) reported a significant improvement in gait symmetry ratio (Kim et al., 2015). Asymmetry in stance and swing time during gait was assessed in one study with no significant improvements reported (Sheikh et al., 2016). Additionally, asymmetry in single support time was assessed in one study with significant improvements observed compared to a control group (Sungkarat et al., 2011). Step length asymmetry was included in two studies (Sungkarat et al., 2011; Sheikh et al., 2016), with only one reporting significant improvements when compared to a control group (Sungkarat et al., 2011). Significant improvements in static (Tung et al., 2010; Sungkarat et al., 2011; Sheikh et al., 2016) and dynamic (Tung et al., 2010; Liu et al., 2016; Sheikh et al., 2016) weight bearing asymmetry were reported in three studies each.

Physical fitness outcomes were assessed in two of the five studies involving resistance exercise; both studies assessed muscular strength for knee extension and flexion, hip extension and flexion, and plantarflexion and dorsiflexion (Tung et al., 2010; Kim et al., 2015). Only one study reported a significant improvement in plantarflexion (Tung et al., 2010), while all other muscular strength measures significantly increased in both studies (Tung et al., 2010; Kim et al., 2015).

Regarding physical function, agility (e.g., Timed-Up-and-Go [TUG]) (Sungkarat et al., 2011), balance (e.g., Berg Balance Scale [BBB], static, and dynamic weight bearing) (Sungkarat et al., 2011; Liu et al., 2016), and lower limb function (e.g., sit-to-stand and time-to-rise) (Tung et al., 2010; Liu et al., 2016) were assessed in one, two, and two of the five studies, respectively. A significant improvement in TUG performance was reported (Sungkarat et al., 2011). Significant between (Sungkarat et al., 2011; Liu et al., 2016) and within group (Tung et al., 2010; Liu et al., 2016) improvements were reported for the BBB in two studies each, while a significant improvement in both static and dynamic balance were reported in one study (Liu et al., 2016). Significant improvements were reported for time-to-rise (Liu et al., 2016) and sit-to-stand performance (Tung et al., 2010).

### Aerobic Exercise Training

Aerobic exercise involved split belt (Lewek et al., 2018) and conventional (e Silva et al., 2017) treadmill walking, while one study involved participants self-selecting the exercise modality (e.g., stationary bike, elliptical machine, or over ground walking) (Escudero-Uribe et al., 2017). Step length asymmetry was assessed in two studies (Escudero-Uribe et al., 2017; Lewek et al., 2018), with significant improvements reported in both trials. Additionally, stance time (Lewek et al., 2018) and swing time (e Silva et al., 2017) asymmetry were each assessed in one study, with no significant improvements reported. None of the three studies assessed physical fitness. However, “comfortable” gait speed (Lewek et al., 2018), BBB (e Silva et al., 2017), and TUG (e Silva et al., 2017) were included in two studies. Significant improvements in both BBB and TUG were reported (e Silva et al., 2017).

### Combined Exercise Training

Only one study incorporated combined lower limb resistance exercise and aerobic exercise (Kim et al., 2017). Gait symmetry ratio significantly improved after training, but no measures of physical fitness were included. No significant improvements were reported on the 10-m walk test.

### Associations

Only one study examined potential associations between changes in asymmetry outcomes and any physical fitness or physical function outcomes (Lewek et al., 2018). A significant, moderate correlation (*r* = −0.45; *p* = 0.04) was observed between change in step length asymmetry and change in gait speed after treadmill walking training. However, the same study reported no significant association between change in stance time asymmetry and change in gait speed (*r* = −0.15; *p* = 0.57).

### Study Quality

The median (interquartile range, IQR) overall TESTEX score was 11 (2), with scores ranging between 9 and 15. The median (IQR) study quality and study reporting scores were 5 (0) and 6 (2), respectively. Overall, studies scored highly for clear specification of eligibility criteria and randomization methods, reporting similar groups at baseline, and blinding of assessors on key outcomes. However, studies consistently scored poorly for not reporting session attendance, not using intention-to-treat analyses, failing to make periodic evidence-based adjustments of exercise intensity, and failing to report all exercise training characteristics (i.e., intensity, duration, frequency, volume, and mode).

## Discussion

The purpose of this review was to evaluate and summarize the effects of exercise training on asymmetries in gait, balance, and physical fitness outcomes in persons with CNCs. Nine RCTs that included a component of exercise training as part of a rehabilitation intervention were retrieved and reviewed. Overall, significant improvements from pre- to post-intervention in the experimental group were observed in four and two studies for gait (Kim et al., 2015, 2017; Escudero-Uribe et al., 2017; Lewek et al., 2018) and balance (Tung et al., 2010; Liu et al., 2016) asymmetry or symmetry outcomes, respectively. While these results are promising, we must be cautious in interpretation given the limited number of studies, lack of consistent results, and other confounding variables (i.e., exercise in combination with other rehabilitation modalities). Herein, each mode of exercise training is evaluated to provide future direction for research and rehabilitation programs involving exercise training to reduce asymmetries in persons with CNCs.

Lower limb resistance exercise was included in the majority of the studies in the current review (Tung et al., 2010; Sungkarat et al., 2011; Kim et al., 2015; Liu et al., 2016; Sheikh et al., 2016). This is not surprising, as previous systematic reviews and meta-analyses have reported positive effects of resistance exercise training on overall muscular strength and power, fatigue, balance, functional capacity, electromyography activity, and quality of life in persons with CNCs (Ada et al., 2006; Motl and Pilutti, 2012; Cruickshank et al., 2015). However, evidence for the effects of resistance exercise on asymmetry outcomes specifically is limited. The current review observed improvements in gait symmetry ratio (Kim et al., 2015) and weight bearing asymmetry (Tung et al., 2010; Sungkarat et al., 2011; Sheikh et al., 2016) after resistance exercise training. Such improvements may result from improved lower limb strength, particularly within the affected limb, allowing more weight to be distributed through the affected limb and generating greater propulsive forces during gait. Indeed, improvements in asymmetry outcomes were paralleled by improvements in lower limb strength in two studies; however, a causal relationship between these variables cannot be assumed (Tung et al., 2010; Kim et al., 2015). Exploration of this potential association is warranted, as previous cross-sectional studies in persons with CNCs and older adults have reported significant negative associations between lower limb strength and power asymmetry, and mobility and balance outcomes (Portegijs et al., 2005; Chung et al., 2008; LaRoche et al., 2012; Larson et al., 2013; Straight et al., 2016; Chon et al., 2018). Future investigations can provide further evidence for the efficacy of resistance training as a rehabilitative strategy to reduce lower limb strength and power asymmetry and explore potential associations with improvements in mobility outcomes. Thus, allowing researchers and clinicians to determine if reductions in lower limb strength and power asymmetry are a potential mechanism for improvements in mobility in persons with CNCs.

Systematic reviews have reported beneficial effects of aerobic exercise training on aerobic fitness, walking speed and endurance, cognitive function, and cardiac disease risk factors in persons with CNCs (Latimer-Cheung et al., 2013; Pang et al., 2013). The current review observed that only step length asymmetry significantly improved following aerobic exercise training (Escudero-Uribe et al., 2017; Lewek et al., 2018). Additionally, a moderate, linear association between change in step length asymmetry and change in “comfortable” gait speed was observed, suggesting that as step length becomes more symmetrical walking speed increases (Lewek et al., 2018). This is in agreement with previous investigations that have observed negative associations between spatiotemporal gait asymmetries and walking speed in persons recovering from stroke (Olney et al., 1994; Kim and Eng, 2003; Balasubramanian et al., 2007; Lewek et al., 2012; Reisman et al., 2013). Differences in step length between the limbs may be due to the affected limb generating lower propulsive forces resulting in a shorter step length for the non-affected limb, and consequently, slower gait speed (Bowden et al., 2006; Balasubramanian et al., 2007). Due to either a lack of reporting or assessing, it is unclear if the aerobic exercise training protocols resulted in improvements in aerobic fitness and walking endurance. This limits the evidence for a potential relationship between changes in step length asymmetry and changes in physical fitness and other mobility outcomes in response to aerobic exercise training. Exploring this relationship warrants future investigations as previous cross-sectional studies have observed significant associations between step length asymmetry and falls in persons recovering from stroke (Sheikh and Hosseini, 2019). A better understanding of this relationship may help to identify changes in gait asymmetry outcomes as potential mechanisms for change in mobility outcomes in persons with CNCs and provide targets for therapeutic interventions.

There is evidence for potential benefits of combined exercise training on mobility and balance outcomes in persons who have experienced a stroke, while one systematic review reported improvements in aerobic capacity, muscular strength, walking speed and endurance, fatigue, and quality of life in persons with MS after combined exercise training (Latimer-Cheung et al., 2013; Saunders et al., 2016). However, neither review reported potentials effects on asymmetry outcomes. The single combined exercise training study in the current review reported a significant improvement in gait symmetry ratio, but this did not appear to translate into improved walking speed (Kim et al., 2017). This was surprising, as cross-sectional analyses in persons recovering from stroke have reported significant associations between asymmetry in gait parameters, and both walking speed and gait energetics (Patterson et al., 2010; Awad et al., 2015). Additionally, significant associations have been reported between reductions in other gait asymmetry parameters (e.g., step length) and increased walking speed in person recovering from stroke after gait training using motor learning strategies to augment asymmetry errors during walking (Lewek et al., 2018).

To the best of our knowledge, a RCT investigating the independent effects of exercise training on asymmetry in gait, balance, and physical fitness outcomes in persons with CNCs is currently lacking. The studies in the current review all incorporated exercise training as part of a comprehensive intervention, and limited our ability to isolate the effects of exercise training from other intervention components. Exercise training has the potential to be an effective rehabilitation modality to reduce asymmetry, due to its adaptability in both prescription and delivery. For instance, previous non-RCTs have prescribed unilateral resistance training for persons with MS to allow for the contralateral limb to produce force independently. Significant improvements in muscular strength were reported in both limbs; however, changes in muscular strength asymmetry were not reported (Broekmans et al., 2011). Also, resistance exercise equipment is readily available at commercial exercise facilities and can be performed at home. Aerobic exercise can also be prescribed in a manner that accommodates asymmetries in lower limb function and gait (Edwards and Pilutti, 2017; Rudroff and Proessl, 2018). Walking can be performed using a split-belt treadmill to allow the lower limbs to travel at different speeds, and this has been used to reduce gait asymmetries in persons with stroke (Reisman et al., 2013). Further, visual feedback during cycling has been reported to reduce asymmetry in force production between the lower limbs in persons with stoke (Ambrosini et al., 2011). However, it must be noted that specialized pieces of equipment, such as a split-belt treadmill, are not available at most commercial and community exercise facilities.

A number of limitations were identified while reviewing the literature. First, only nine studies met the inclusion criteria, highlighting the need for more RCTs of exercise training on asymmetry in persons with CNCs. Although there is evidence to support the presence of asymmetry in physical fitness outcomes (i.e., muscular strength) among persons with CNCs, only asymmetry outcomes pertaining to gait and balance were reported in the included studies (Rudroff and Proessl, 2018). Eight of the nine reviewed RCTs included persons with stroke, limiting the ability to evaluate the efficacy of exercise training on asymmetry outcomes in other CNCs. An important limitation is the consistent lack of reporting of exercise prescription (i.e., frequency, duration, intensity, and exercise selection). For example, when lower limb resistance exercise was included, no details were provided pertaining to the number of repetitions and sets, rest interval duration, and the specific exercises performed. Further, the exercise training may not have been prescribed in a manner to specifically target and reduce asymmetry, and its individual contribution to asymmetry outcomes cannot be isolated given the combined rehabilitation interventions. Another important limitation to the literature was the variability in the asymmetry outcomes included and the method of calculation. A call for the standardization in the calculation of asymmetry outcomes in research in persons with stroke has been made to aid in the interpretation of results between studies (Patterson et al., 2010). This standardization should be applied to all research involving persons with CNCs. In addition to the limitations of the literature, there are also limitations to the review itself. Included studies were limited to RCTs, published in English academic journals, and involved a component of exercise training. Studies were reviewed and selected by two members of the research team, and were therefore subject to selection bias.

There is limited, high-quality evidence for the effect of exercise training on gait, balance, and physical fitness asymmetry in persons with CNCs. This review summarizes the current, preliminary literature, which suggests the potential of exercise training to improve gait and balance asymmetry in this population. However, due to the limited number of studies and confounding variables, these findings must be interpreted with caution. Future investigations are needed to address the limitations of the current evidence, particularly regarding the independent effects of exercise training on asymmetry, and the inclusion of asymmetry outcomes related to physical fitness in persons with CNCs. Such future research will provide much needed evidence on the potential of exercise training for managing asymmetry in persons with CNCs.

## Data Availability Statement

The original contributions presented in the study are included in the article/supplementary material, further inquiries can be directed to the corresponding author/s.

## Author Contributions

JF and LP developed the concept for this review and designed the study. Data collection and extraction, and analysis was completed by JF and JM. Results were interpreted by JF, JM, and LP. Manuscript was written by JF and JM, and edited by LP. All authors approved the final manuscript.

## Conflict of Interest

The authors declare that the research was conducted in the absence of any commercial or financial relationships that could be construed as a potential conflict of interest.
